# Germline Mutation in *NLRP2* (*NALP2*) in a Familial Imprinting Disorder (Beckwith-Wiedemann Syndrome)

**DOI:** 10.1371/journal.pgen.1000423

**Published:** 2009-03-20

**Authors:** Esther Meyer, Derek Lim, Shanaz Pasha, Louise J. Tee, Fatimah Rahman, John R. W. Yates, C. Geoffrey Woods, Wolf Reik, Eamonn R. Maher

**Affiliations:** 1 Department of Medical and Molecular Genetics, Institute of Biomedical Research, University of Birmingham, Birmingham, United Kingdom; 2 West Midlands Regional Genetics Service, Birmingham Women's Hospital, Edgbaston, Birmingham, United Kingdom; 3 Department of Medical Genetics, University of Cambridge, Cambridge, United Kingdom; 4 Institute for Medical Research, Addenbrooke's Hospital, Cambridge, United Kingdom; 5 East Anglian Medical Genetics Service, Addenbrooke's Treatment Centre, Addenbrooke's Hospital, Cambridge, United Kingdom; 6 Laboratory of Developmental Genetics and Imprinting, The Babraham Institute, Cambridge, United Kingdom; 7 Centre for Trophoblast Research, University of Cambridge, Cambridge, United Kingdom; Stanford University School of Medicine, United States of America

## Abstract

Beckwith-Wiedemann syndrome (BWS) is a fetal overgrowth and human imprinting disorder resulting from the deregulation of a number of genes, including *IGF2* and *CDKN1C*, in the imprinted gene cluster on chromosome 11p15.5. Most cases are sporadic and result from epimutations at either of the two 11p15.5 imprinting centres (IC1 and IC2). However, rare familial cases may be associated with germline 11p15.5 deletions causing abnormal imprinting *in cis*. We report a family with BWS and an IC2 epimutation in which affected siblings had inherited different parental 11p15.5 alleles excluding an *in cis* mechanism. Using a positional-candidate gene approach, we found that the mother was homozygous for a frameshift mutation in exon 6 of *NLRP2*. While germline mutations in *NLRP7* have previously been associated with familial hydatidiform mole, this is the first description of *NLRP2* mutation in human disease and the first report of a *trans* mechanism for disordered imprinting in BWS. These observations are consistent with the hypothesis that *NLRP2* has a previously unrecognised role in establishing or maintaining genomic imprinting in humans.

## Author Summary

A small set of genes (imprinted genes) are expressed in a “parent-of-origin” manner, a phenomenon known as genomic imprinting. Research in human disorders associated with aberrant genomic imprinting provided insights into the molecular mechanisms of genomic imprinting and the role of imprinted genes in normal growth and development. Beckwith-Wiedemann syndrome (BWS) is a congenital overgrowth syndrome associated with developmental abnormalities and a predisposition to embryonic tumours. BWS results from alterations in expression or function of imprinted genes in the imprinted gene cluster at chromosome 11p15. Although BWS may be caused by a variety of molecular mechanisms, to date, all the genetic and epigenetic defects associated with BWS have been limited to 11p15.5. We report a family with two children affected with BWS and an epigenetic defect at 11p15.5 in which the primary genetic defect mapped outside the imprinted gene cluster. Using autozygosity mapping, we found an extended homozygous region on chromosome 19q13.4 (containing *NLRP2* and *NLRP7* genes) in the mother. Homozygous inactivating mutations in *NLRP7* in women have been associated previously with abnormal imprinting and recurrent hydatidiform moles. We identified a homozygous frameshift mutation in *NLRP2* in the mother of the two children with BWS implicating *NLRP2* in the establishment and/or maintenance of genomic imprinting/methylation.

## Introduction

Genomic imprinting is an epigenetic modification that causes genes to be expressed according to their parent of origin. Although less than 100 imprinted genes have been identified in human and mice, many imprinted genes appear to have a critical role in prenatal growth and development [1]. Molecular genetic analysis of rare human imprinting disorders has played a critical role in elucidating the mechanisms of genomic imprinting. In particular, studies of the imprinting disorder Beckwith-Wiedemann syndrome (BWS MIM 130650) have provided important insights into the structure and function of imprinting centres [2]. BWS is a congenital overgrowth syndrome, characterised by prenatal and postnatal overgrowth, macroglossia and anterior abdominal wall defects. Additionally, variable features include organomegaly, neonatal hypoglycaemia, hemihypertrophy, urogenital abnormalities and in about 5% of children, embryonal tumours (most frequently Wilms' tumour). The genetics of BWS are complex, but involve mutation or altered expression of several closely linked genes associated with cell cycle and growth control in the imprinted 11p15.5 chromosomal region. Imprinted genes most frequently implicated in the aetiology of BWS include the paternally expressed *IGF2, KCNQ1OT1* (*LIT1*) genes and the maternally expressed *H19* and *CDKN1C (P57KIP2)* genes. *KCNQ1OT1* and *H19* transcripts are not translated but the *IGF2* gene product is an important prenatal growth factor and the CDKN1C protein is a candidate tumour suppressor that negatively regulates the cell cycle [3]. The majority of BWS cases are sporadic and result from epimutations of the distal (IC1) or proximal (IC2) 11p15.5 imprinting centres (see [4] and references within). IC1 is a differentially methylated region (DMR) about 5kb upstream of *H19* that has an “insulator function” regulated by the zinc finger transcription factor, CTCF. The insulator is methylation sensitive, such that when CTCF binds to the unmethylated maternal allele, the *IGF2* promoters do not have access to (are insulated from) enhancers downstream of *H19*. Methylation on the paternal allele prevents CTCF from binding, thus permitting interaction between the *IGF2* promoters and the enhancers [5]. About 5–10% of sporadic BWS cases have hypermethylation of the *H19* DMR and in these cases *IGF2* shows loss of imprinting (LOI) and biallelic expression [6]. The second imprinting centre, IC2 is a DMR located in intron 10 of the *KCNQ1* gene and is known as KvDMR1. The unmethylated paternal allele permits transcription of the antisense transcript *KCNQ1OT1* (also known as *LIT1*) and silencing of genes including *KCNQ1* and *CDKN1C*. Maternal methylation at the KvDMR1 is thought to prevent transcription of the *KCNQ1OT1* gene and enable expression of *CDKN1C*. Loss of methylation (LOM) at the KvDMR1 is seen in up to 50% of sporadic BWS and is associated with biallelic expression (loss of imprinting) of *KCNQ1OT1* and silencing of maternal *CDKN1C* expression [7]–[9]. Apparent hypomethylation of IC2 may, in rare cases, result from a germline IC2 deletion [10]. However, most BWS patients with IC2 methylation defects appear to have an epimutation of unknown cause (although there is an increased risk of BWS with IC2 epimutation in children conceived by assisted reproductive technologies) [11]–[13]. In order to gain insights into the factors responsible for IC2 imprinting defects, we studied a family with BWS that displayed evidence of IC2 epimutations through a *trans* mechanism.

## Materials and Methods

### Ethics Statement

This study was conducted according to the principles expressed in the Declaration of Helsinki. The study was approved by the South Birmingham Research Ethics Committee (equivalent to the Institutional Review Board of Birmingham Women's Hospital) reference number CA/5175. All patients provided written informed consent for the collection of samples and subsequent analysis.

### Study Subjects

A consanguineous family of Pakistani origin with two affected children with BWS due to loss of methylation at KvDMR1 were investigated in the first instance. Following the identification of *NLRP2* mutations in this family, a further 11 BWS families, each with a single case of BWS (mean age 10.8 years) with KvDMR1 loss of methylation were analysed for *NLRP2* mutations. This cohort included 10 patients who also had loss of methylation at other imprinted loci. Ethnically matched laboratory control samples were analysed to evaluate the significance of novel sequence variants.

### Molecular Genetic Studies

Genomic DNA was extracted from peripheral lymphocytes by standard techniques.

In preliminary examinations chromosomal abnormalities were excluded and the methylation status of IC1 and IC2 of imprinting region 11p15.5 were determined.

Methylation analysis of KvDMR1 was performed as described previously, with PCR amplification of bisulphite modified DNA and digestion with restriction enzyme *Bst*U1 yielding different sized fragments which is separated using ABI377 or 3730 [4]. In addition, methylation status at 3 additional DMRs was evaluated at the Transient Neonatal Diabetes Mellitus (TND) locus at 6q24, 7q32 *(PEG1*) and the Angelman/Prader-Willi locus at 15q13 (*SNRPN*) as described previously [14]. Primers and methods for the analysis of the methylation status of PEG1 DMR by methylation specific PCR (MS-PCR) were obtained from previously published report by Mackay et al [15].

For linkage studies a genome wide linkage scan was undertaken using the Affymetrix 250k SNP microarray. Mutation analysis of *JMJD2D*, *ZFP57*, *NLRP7* and *NLRP2* was carried out by direct sequencing. The genomic DNA sequence of these genes was taken from Ensembl (http://www.ensembl.org/index.html) and primer pairs for the translated exons were designed using primer3 software (http://fokker.wi.mit.edu/primer3/input.htm). Amplification was performed according to standard protocols with Bio Mix Red provided by Bioline. PCR products were directly sequenced by the Big Dye Terminator Cycle Sequencing System with the use of an ABI PRISM 3730 DNA Analyzer (Applied Biosystem). DNA sequences were analyzed using Chromas software.

## Results

### Case Report

**Figure 1:**
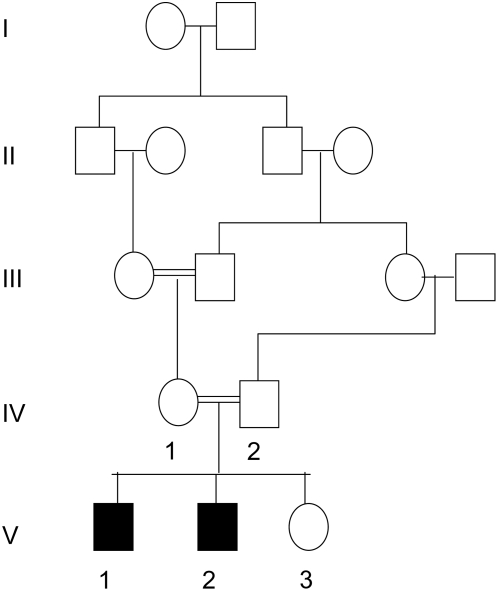
Family pedigree (partial) to demonstrate complex consanguinity.

A family with complex consanguinity (Figure 1) was ascertained after the diagnosis of two children with BWS. Both pregnancies were complicated by polyhydramnios and raised hHCG levels. Both affected children were born by Caesarean section. At birth Child 1 (V-1 in Figure 1) was macrosomic (4.55 kg at 38 weeks gestation) and was noted to have macroglossia, an omphalocele, ear creases, right inguinal hernia, undescended testis and neonatal hypoglycaemia in the first two days after birth. Similarly Child 2 (V-2) was macrosomic (3.633 kg at 35 weeks gestation) with macroglossia, ear lobe creases and neonatal hypoglycaemia that was difficult to control. Subsequently a third child was born without features of BWS (between the second and third children a probable hydatidiform mole was diagnosed). Molecular studies demonstrated loss of maternal allele KvDMR1 (IC2) methylation in both affected children, but the unaffected sibling had normal methylation. H19 methylation status was normal in both affected children and MLPA analysis demonstrated no evidence of an IC1 or IC2 deletion. Genotyping revealed no evidence of paternal uniparental disomy and linkage analysis with microsatellite markers flanking IC2 (TH and D11S4088) demonstrated that the two children had inherited opposite maternal and paternal 11p15.5 alleles. These findings were consistent with IC2 epimutation resulting from a *trans* imprinting defect.

### Molecular Genetic Analysis

**Figure 2:**
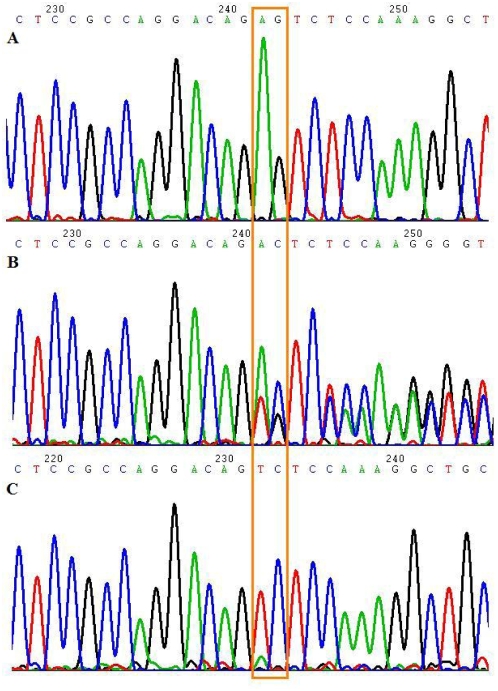
Germline *NLRP2* mutation. The alignments of *NLRP2* nucleotides c.1465–c.1492 are shown. (A) is wildtype sequence in a control, (B) is heterozygous *NLRP2* mutation (c.1479delAG) in the father, and (C) is homozygous 2bp deletion (c.1479delAG) in the mother.

In view of the history of consanguinity, an autosomal recessive disorder was suspected (either affecting both children or affecting the mother). Genetic linkage studies were undertaken by genotyping the two children and both parents on an Affymetrix 250k SNP array platform. Five regions of homozygosity (>2Mbases) were shared by the two children but these did not contain a gene known to be implicated in the establishment or maintenance of genomic imprinting. However inspection of the maternal genotypes revealed an ∼8 Mbase homozygous region containing *NLRP2* and *NLRP7* at 19q13.4. *NLRP7* is a homologue of the mouse *NLRP2* gene (*NLRP7* is not present in the mouse) and the human *NLRP2* gene. Sequencing of *NLRP2* in the mother identified a homozygous frameshift mutation ((c.1479delAG, NM_017852; Figure 2) that was predicted (in the absence of nonsense-mediated RNA decay) to result in a truncated protein (p.Arg493SerfsX32) lacking 539 amino acids from the C-terminal that includes the LRR domain. The mutation was not detected in 542 ethnically matched control chromosomes but the father was heterozygous for the mutation, child 1 was homozygous for the mutation and the other two children were heterozygous. Mutation analysis of 11 additional families with BWS did not reveal any evidence of pathogenic *NLRP2* mutations.

**Figure 3:**
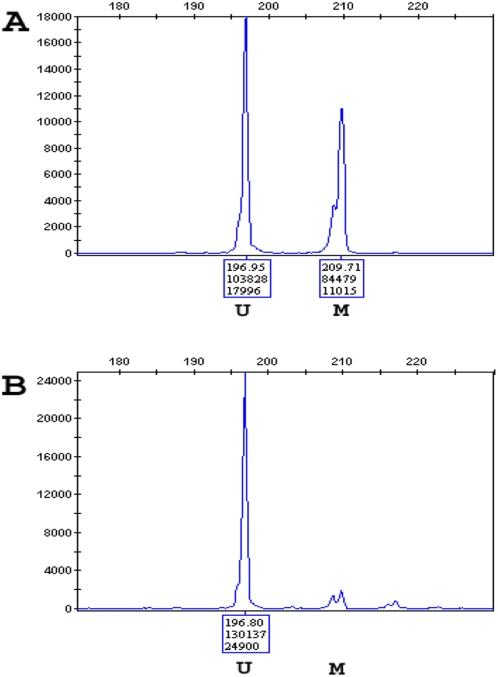
Loss of methylation at *PEG1* DMR. Electropherogram of amplification products of MS-PCR. U, unmethylated product; M, methylated product. The X-axis represents the calculated product size (in bp and also represented as the top number in the box). The Y axis represents the peak height (bottom number in the box). The methylated to unmethylated ratio was calculated as the area under the curve (middle number in the box) of methylated versus unmethylated amplified products. (A) Normal Control (ratio 0.81), (B) Child 2 with LOM at *PEG1.*

To determine if the *trans* imprinting defect extended beyond KvDMR1, we analysed methylation levels at the TND (6q24), *SNRPN* (15q13) and *PEG1* (7q32) DMRs. Both affected siblings (and all controls) had normal methylation levels at the TND and *SNRPN* DMRs but Child 2 demonstrated partial loss of methylation at the *PEG1* DMR (Figure 3).

## Discussion

We identified a homozygous frameshift mutation in the mother of two children with BWS caused by epimutations at IC2 (KvDMR1). Most cases of BWS due to loss of methylation of KvDMR1 are sporadic, but a handful of familial cases have been described with maternally inherited germline IC2 deletions [10]. However, in our family there was no evidence of a germline deletion by MLPA analysis [16] and the two affected children were shown to have inherited opposite maternal and paternal 11p15.5 alleles. This suggested that in this family, KvDMR1 LOM resulted from a *trans* and not a *in cis* effect. Germline *NLRP2* mutations have not been reported previously, but mutations in *ZFP57* and *NLRP7* can cause imprinting disorders in which epimutations at imprinted loci result from a *trans* effect. Thus individuals homozygous for *ZFP57* mutations presented with transient neonatal diabetes mellitus (TNDM). The major cause of TNDM is aberrant expression of imprinted genes at chromosome 6q24 and about 20% of cases have LOM at the TND differentially methylated region (DMR). Patients with homozygous *ZFP57* mutations have LOM at the TND DMR, but also at other imprinted loci including KvDMR1 [15]. However, there was no evidence of germline *ZFP57* mutations in our family. Germline *NLRP7* mutations are associated with familial recurrent biparental complete hydatidiform mole (FHM) in which there is epigenetic abnormalities at DMRs in multiple imprinting regions [17]–[19]. Although FHM associated with *NLRP7* mutations is inherited in an autosomal recessive manner, in contrast to *ZFP57* mutation, homozygotes have normal genomic methylation but in female homozygotes there is a failure to establish methylation imprints in their germ cells leading to hydatidiform moles and reproductive wastage (male homozygotes do not have imprinting defects in their sperm). The methylation defects in FHM are specific for imprinted loci, and DNA methylation at non-imprinted genes and genes subject to X-inactivation is unaffected [18]. The human *NLRP2* and *NLRP7* genes are highly homologous and the two proteins consist of 1,062 and 1,009 amino acids respectively and have identical structure and about 64% amino acid identity. Only one of the two affected children with BWS was homozygous for a *NLRP2* mutation and, by analogy with FHM caused by *NLRP7* mutations, the BWS phenotype most likely results from homozygosity in the mother, such that familial BWS associated with *NLRP2* mutations is inherited in a similar manner to *NLRP7* associated FHM and not in a conventional autosomal recessive manner.

Both FHM and *ZFP57*–TNDM are associated with imprinting aberrations at multiple loci, and we identified partial loss of methylation at the *PEG1* DMR in one of the affected children. Nevertheless it seems that *NLRP2* mutations have a less severe effect on imprinting than *NLRP7* or *ZFP57* inactivation. A subset of children with BWS and an IC2 epimutation display hypomethylation at multiple imprinting centres (DMRs) (in our series these children are more likely to have been conceived by assisted reproductive technologies) [14],[20]. To our knowledge, none of these cases have been familial and we did not identify *NLRP2* mutation in the sporadic cases we studied. It appears that the establishment (or maintenance) of methylation at KvDMR1 is particularly sensitive to genetic and/or environmental insults. We note that one of the affected children demonstrated a partial loss of methylation at *PEG1* DMR1 both by bisulphite sequencing and MS-PCR suggesting that *NLRP2* mutations may be associated with an incomplete failure of imprinting establishment and/or a partial failure of maintenance methylation at this DMR. Interestingly, investigation of a mouse knockout of *ZFP57* has suggested a role in both the establishment of germline methylation imprints and in the postfertilisation maintenance of methylation imprints [21].

*NLRP2* and *NLRP7* encode members of the *NLRP* (Nucleotide-binding oligomerization domain, Leucine rich Repeat and Pyrin domain) family of CATERPILLER proteins. NLRP family of cytoplasmic proteins comprises 14 members of similar structure that are principally encoded by two gene clusters on chromosome 11p15 (*NLRP*6, 10 and 14) and 19q13.4 (*NLRP*2, 4, 5, 7, 8, 9, 11, 12 and 13). Most of the family members are well conserved from *C. elegans*, *D. melanogaster*, rat, and mouse to human but there is no rodent homologue for *NLRP7* and the gene is found in only a few genomes (human, primate and cow). Some NLRP proteins are components of the inflammasome that is implicated in the sensing of, and inflammatory reaction to, extracellular pathogens and intracellular noxious compounds [22]. Germline mutations in *NLRP3* and *NLRP12* are associated with familial cold autoinflammatory syndrome [23],[24]. NLRP2 was suggested to function as a modulator of macrophage NFKB activation and procaspase 1 [25], however we found that the two family member homozygous for a *NLRP2* truncating mutation did not show any evidence of an immune or autoinflammatory disorder. Nevertheless most NLRP family proteins are widely expressed and not restricted to the immune system. In addition, many are expressed in human oocytes and embryos at an early stage of development. Thus Zhang et al. have reported that *NLRP*4, 5, 8, 9, 11, 12, 13, and 14 were highly expressed in oocytes and then gradually decreased in embryos with a very low level in day 5 embryos, whilst *NLRP2* and *NLRP*7 progressively decreased from oocytes to day 3 embryos then showed a sharp increase on day 5 [26]. These observations are consistent with *NLRP2* and *NLRP*7 having a similar role in early development/imprinting establishment. Although it has been suggested that FHM might result from an immune-related defect in oogenesis or early embryo development (with methylation changes being a secondary phenomenon) the specific association of the methylation defects with imprinted DMRs suggests a more direct role in the establishment or maintenance of imprinting marks. Such a view is supported by the identification of germline *NLRP2* mutations in BWS and should prompt further investigation of the role of *NLRP2* and *NLRP7* in genomic imprinting. The apparent involvement of NLRP proteins in genome methylation and the sensing and inflammatory response to extracellular pathogens and intracellular noxious compounds is intriguing given the suggestion that cytosine methylation may have evolved as a host response to transposons [27]. It is interesting that the third child in the family we report was unaffected. Most mothers with *NLRP7* mutations have recurrent molar pregnancies only, but at least three families have been reported in which affected women had liveborn offspring (see [28] and references within). In one family, three affected members had, in addition to the molar phenotype, several miscarriages and three term pregnancies [29]. In one of the term pregnancies the baby was born with severe intrauterine growth retardation, but grew into a healthy adult with normal methylation levels [30]. In another term pregnancy the baby was born with unilateral cleft lip and palate and later manifested idiopathic delayed mental and motor development [31]. Murdoch et al. detected a homozygous splice site mutation in *NLRP7* in all three affected women of this family [32]. Thus homozygous *NLRP7* mutations may be associated with clinical heterogeneity/incomplete penetrance, possibly resulting from genetic modifier or environmental effects. In the light of these observations and the apparently milder phenotypic effects of maternal *NLRP2* inactivation than *NLRP7* inactivation (Beckwith-Wiedemann syndrome and molar pregnancy respectively) it might be predicted that clinical heterogeneity/incomplete penetrance would be a feature of maternal *NLRP2* inactivation. Although maternal *NLRP2* mutations appear to be a rare cause of familial BWS, the identification of these cases is important, as the inheritance pattern differs from the autosomal dominant inheritance (with parent of origin effects) associated with other inherited forms of BWS. The inheritance of *NLRP2*-associated BWS has similarities to other autosomal recessive disorders in which homozygous mothers are well, but there is a high risk to their offspring (e.g. FHM and treated maternal phenylketonuria).
